# A human rights-based analysis of the experiences and challenges faced by children with albinism and their mothers: Insights from the African film ‘Can You See Us?’

**DOI:** 10.4102/ajod.v15i0.1791

**Published:** 2026-04-30

**Authors:** Priccilar Vengesai, Maureen Mswela

**Affiliations:** 1 Department of Jurisprudence, College of Law, University of South Africa, Pretoria, South Africa

**Keywords:** albinism, mothers, children, Africa, human rights, film

## Abstract

**Background:**

This study utilises the cinematic work ‘Can You See Us’ as a prism to elucidate contemporary discourse surrounding the intricate tribulations confronted by children with albinism and their mothers. Despite increasing academic research on the connection between albinism and motherhood experiences, meaningful critical discussions on this topic remain inadequately addressed within both scholarly frameworks and African policy and human rights paradigms.

**Objectives:**

Utilising a human rights-based approach, the inquiry elucidates how systemic sufferings besiege a child with albinism and his mother. It also reveals that the challenges endured by children with albinism and their mothers are not merely individual forays but are intricately interwoven within expansive socio-political matrices that perpetuate inequity and discrimination.

**Method:**

A mixed qualitative methodology was employed, incorporating film observation and document-based analysis.

**Results:**

The findings of this study illuminate profound adversities confronted by individuals afflicted with albinism, encompassing destitution, experiences of corporeal and psychological maltreatment, incapacity to participate in normative social interactions, bullying, abuse and violent assaults, which can, in the most grievous of circumstances, culminate in dire outcomes.

**Conclusion:**

We conclude that the predicaments depicted in the film are pervasive across the African expanse. These challenges ought to be recognised as transgressions against intrinsic human rights. Importantly, the extant regional action plan addressing albinism in Africa neglects the integration of a thorough gender-sensitive paradigm, especially concerning the nuances of motherhood and its confluence with albinism.

**Contribution:**

This cinematic analysis offers a transformative paradigm for apprehending the experiences of mothers nurturing children with albinism within the African milieu.

## Introduction

Albinism is an inherited condition characterised by a conspicuous deficiency in melanin biosynthesis (Punaks, Baker & Miti-Drummond 2024:12; Taylor & Lund 2008:1249). This autosomal recessive trait necessitates that both progenitors be carriers of the albinism allele for phenotypic expression in offspring (Ngula 2023:432). Oculocutaneous albinism, the most prevalent phenotype observed in Africa, is delineated by a partial or complete absence of melanin pigmentation across skin, hair and ocular tissues (Punaks et al. 2024:12; Reimer-Kirkham et al. 2024:1; Taylor et al. 2021:1). The global incidence of albinism is estimated between 1 in 1000 and 1 in 15 000; however, the African prevalence is markedly elevated, ranging from 1 in 1500 to 1 in 5000 (Likumbo, De Villiers & Kyriacos 2021:1; Reimer-Kirkham et al. 2024:1; Taylor et al. 2021:1). International discourse has unequivocally classified albinism as a disability (Hargovan & Chetty 2023:155; Likumbo et al. 2021:2) with acknowledgement in certain African jurisdictions, including South Africa (Mswela 2018:2). The physiological manifestations of albinism predispose affected children to significant visual impairments and pronounced vulnerability to ultraviolet-induced dermal damage (Taylor & Lund 2008:1249), conditions that are exacerbated by prolonged solar exposure and inadequate healthcare, particularly in rural locales across Africa (Mswela 2022:27–28; Taylor & Lund 2008:1249).

Despite the extensive ramifications of albinism affecting countless individuals in Africa, there exists a pervasive ignorance surrounding the condition (Africa Albinism Network & Albinism Multipurpose Organisation n.d.; Chagutah 2025:6). Cultural paradigms, superstitions and entrenched mythologies profoundly shape societal perceptions of albinism within diverse African communities (Chagutah 2025:6; Chirwa & Kalaluka 2025:1). Erroneous beliefs often characterise albinism as a malediction or a form of contamination (Mswela 2025:27). Such pernicious beliefs result in considerable stigma, subjecting individuals with albinism to multifarious forms of discrimination and human rights violations (African Court on Human and Peoples’ Rights, 2025:4). The systemic marginalisation of this demographic epitomises a significant human rights dilemma, necessitating an intricate and comprehensive strategy (Mswela 2025:27). Within various African nations, the dearth of targeted legal safeguards for individuals with albinism exacerbates their exposure to discrimination and violence. From a motherhood standpoint, rearing a child with albinism often entails profound social isolation, financial strain and emotional turmoil, particularly in contexts where structured support mechanisms are severely lacking or altogether absent (Buyco et al. 2025:12; Reimer-Kirkham et al. 2021:725; Strobell 2020:105).

To elucidate the formidable adversities encountered by children with albinism and their mothers, the cinematic work *Can You See Us?* (Netflix 2022), set against the backdrop of Zambia, emerges as a salient case study.

Despite ongoing academic discourse encapsulating that highlights the tribulations of these individuals (Hargovan & Chetty 2023; Reimer-Kirkham et al. 2021; Taylor et al. 2021), a holistic dialogue encompassing the collective narratives of children with albinism and their mothers remains lamentably deficient. This study undertakes an incisive film analysis to unveil the multifaceted challenges beleaguering these vulnerable demographics across the African continent. Film analysis offers a nuanced understanding of the lived experiences that are central to our inquiry.

In this article, we provide a synopsis of the film and findings pertinent to the myriad adversities faced by the child with albinism and corroborate these insights with relevant literature. We theorise these tribulations through a human rights-based approach, concluding that the predicaments faced are unequivocal violations of fundamental human rights. The penultimate section offers recommendations for establishing houses for children with albinism and their maternal caretakers, providing psychosocial counselling services for these mothers, organising regional symposia to raise awareness about albinism and developing specific regional protocols tailored for individuals with albinism and their mothers. Our prioritisation of mothers in these recommendations is reinforced by consistent findings of their disproportionate burden of stigma and their caregiving roles in African contexts, as poignantly illustrated in *Can You See Us?* (Netflix 2022) and supported by extant scholarship (Buyco et al. 2025:12; Reimer-Kirkham et al. 2021:725; Strobell 2020:105).

Nevertheless, the conspicuous neglect of paternal experiences underscores an urgent call for more inclusive and expansive research in the future.

### Theoretical framework

This study investigates the myriad challenges encountered by children with albinism and their mothers, employing a human rights-based approach. As elucidated by Broberg and Sano (2017:669), a human rights-based approach is fundamentally tethered to international human rights frameworks and is dedicated to the promotion and safeguarding of human rights, rooted in principles such as intrinsic dignity, equality and non-discrimination (Chacon-Hurtado et al. 2024:901). The global origin of this approach is reflected in the Universal Declaration of Human Rights (United Nations 1948), while various African human rights instruments reinforce these core tenets (Chacon-Hurtado et al. 2024:901).

In adopting a human rights-based lens, this study recognises both children with albinism and their mothers as legitimate rights holders, diverging from the traditional disability rights model that emphasises the entitlements of people with disabilities (Pane & Yanis 2023:148). The disability model sidelines the rights and experiences of caregivers, particularly mothers. While children with albinism suffer from numerous human rights abuses, their mothers inevitably encounter increasing violations. The human rights-based approach acknowledges the necessity of protecting the rights of both children with albinism and their mothers. Thus, this approach opens a discussion for the need for a comprehensive strategy for addressing the rights of these interconnected groups, as depicted in the film ‘Can You See Us?’, thereby confronting and rectifying the human rights infringements faced by these two vulnerable demographics in Africa. By employing a human rights-based approach, this study underscores the urgent necessity to maintain these marginalised groups as rightful subjects of rights.

Human rights-based approach has been used in research about mothers who are impacted with albinism (Reimer-Kirkman et al. 2021:285) and children with albinism separately (Ero et al. 2021:7). A holistic approach to both proves that they go through the same societal agony, hence a holistic solution must be sought. The five pillars of human rights-based approach, non-discrimination, accountability, participation, empowerment and egality lead to the reduction of challenges and human rights violations faced by children with albinism and their mothers (Ero et al. 2021:7). The approach assets that the rights of children with albinism and their mothers must be promoted and protected because they are rights holders like any other member of the community.

Ultimately, by promoting the principle of participation, the human rights-based approach aims to empower people with albinism and their mothers to claim their rights and hold states accountable for responding to such claims. African states’ responses to these human rights claims must address the unique challenges faced by these vulnerable groups.

## Research methods and design

This study utilised an eclectic qualitative methodology, incorporating both filmic observation and document-based analysis. Initially, we selected *Can You See Us?* (Netflix 2022), for its authentic and nuanced portrayal of the challenges faced by a child with albinism and his mother in the Southern African milieu. The selection criteria were predicated on its cultural pertinence, narrative emphasis on the corporeal and psychosocial adversities encountered by individuals children with albinism and their mothers, and its established role as an advocacy instrument for social inclusion and awareness. Following the film selection, a systematic, in-depth viewing process was conducted in the first phase to distil key themes aligned with the study objectives. We meticulously analysed the audio, including subtitles and the actors’ dialogue. A rigorous observational methodology was employed, focusing on both verbal discourse and non-verbal expressiveness. Specific excerpts were meticulously chosen for their representational power concerning the challenges confronted by the child and his mother, each excerpt annotated with playback timestamps for precise traceability. We used the film’s character identities as a guide for our references to the excerpts in the findings. Non-verbal comportments, including gestures and facial expressions, were carefully scrutinised to expose underlying prejudicial attitudes directed towards the child with albinism and his mother.

To further elucidate the meanings imbued in the film, we employed Roland Barthes’s semiotic analysis framework, delineating both denotative and connotative significations from selected extracts (Rais & Fadillah 2025:2). Denotative meanings were gleaned from explicit content, such as dialogue addressing discrimination. In contrast, connotative meanings were derived from symbolic elements, such as visual motifs and interpersonal dynamics that mirror societal perceptions of albinism. The observational phase included repeated viewings of the film to validate findings and ensure exhaustive data accumulation.

Subsequently, an extensive literature review was conducted to enrich and contextualise the film’s observed findings within the framework of existing scholarship. The literature was deliberately sampled through systematic analysis to uphold relevance to the research objectives, drawing upon African Union human rights instruments, case law, legislation and secondary sources. Secondary sources were accessed via the Unisa E-library and physical repositories, including academic journals, textbooks and book chapters, sourced through databases such as ProQuest, ScienceDirect and ScienceLO. Strategic Google searches facilitated access to news articles and various reports that provided quantitative support to augment the discourse.

### Ethical considerations

Ethical clearance to conduct this study was obtained from the University of South Africa, College of Law, Research Ethics Review Committee (No. 8142).

## Results

### Film summary

‘Can You See Us?’ is a 2022 Zambian film grounded in real events, revolving around the life of a Zambian musician, and is available on Netflix (Johnson 2023; Netflix 2022). The narrative commences with the mother (Chama) portraying pregnancy, depicting the support offered by her husband, Kennedy, as they await the birth of their son, Joseph. However, upon Joseph’s birth and the subsequent revelation of his albinism, Kennedy reacts with disdain towards Chama, blaming her for their child’s disability, leading to his abandonment of both at the hospital. Upon their release, Chama and Joseph return home only to discover their eviction was orchestrated by Kennedy. Chama then enters into a marital union with Martin, the taxi driver who was hired to transport her and Joseph from Kennedy’s residence. Within this new marital union, Chama resolves against bearing additional children, propelled by the fear of conceiving another child with albinism. To safeguard Joseph from anticipated external dangers, she opts for home-based schooling, significantly curtailing his mobility. When Joseph eventually integrates into a community and attends formal schooling, he endures recurrent episodes of hostility from his peers. His predicament intensifies when a business person becomes fixated on him, mistakenly believing that Joseph’s albinism can bring a financial fortune for his businesses.

Following the tragic passing of Chama and Martin, Joseph is thrust into the care of Kennedy and his spouse, Jennifer, who harbours animosity towards him, further exacerbating his precarious situation.

### Challenges faced by children with albinism and their mothers

The documentary illuminates the myriad adversities faced by children with albinism and their mothers, encompassing displacement, abuse, bullying, systemic discrimination and a threat to life. While inevitable adversities resonate on both sides, others are distinctly encountered by either the child or the mother, as elucidated below.

### Displacement from home

Chama’s giving birth to her son, Joseph, who was born with albinism, culminated in her expulsion from her matrimonial home, orchestrated by her husband, Kennedy, and her mother-in-law, hereinafter referred to as Mama. Her harrowing journey commenced with abandonment at the hospital, and upon her return post-discharge, it became apparent that her presence was no longer welcome. Mama unequivocally articulated her intentions, saying, ‘I don’t want you in my house anymore’ (Mama [Netflix 2022:01:41:55]). Additionally, Kennedy forcibly evicted Chama and Joseph from their home and even hired a taxi to facilitate their exit. The visuals show a Kennedy hastily packing Chama’s belongings into a bag, while she frantically shows a determination to retain her place in both her home and her marriage. In a moment thick with tension, Kennedy issued a threat of physical violence, saying, ‘I will hit you’ (Kennedy [Netflix 2022:01:35:47]). The film depicts Chama’s coerced; her belongings were hurled outside the house. Amid this turmoil, Mama directed Kennedy’s sibling, Pheli, to carry baby Joseph from the house (Mama [Netflix 2022:01:34:11]). This orchestrated ousting, marked by a collusion among Mama, Kennedy, and Pheli, underscores that Chama and Joseph did not merely relinquish their domicile but were also bereft of family support at a perilous moment. The evidence presented reveals a profoundly ingrained pattern of prejudicial treatment, underscored by familial violence, which has a direct and deleterious impact on both the mother and her child with albinism.

### Physical and psychological abuse

This analysis contends that children with albinism and their mothers endure both corporeal and psychological maltreatment, perpetrated not solely by immediate kin but also by societal constituents. Such dynamics are illustrated in the film *Can You See Us* (Netflix 2022), with portrayals corroborated by extensive empirical research (Mtonga et al. 2025:74) and scholarly treatises (Benyah 2017:161). The characters Chama and Joseph suffer abuse at the hands of Kennedy, who, upon discerning Joseph’s disability, renounces his paternity, exclaiming, ‘Whatever that thing is, it’s not my child!’ (Kennedy [Netflix 2022:01:42:37]). This exclamation inflicts psychological trauma upon Chama, compelling her to shoulder the blame linked to bearing a child with albinism, while simultaneously manifesting an early rejection of Joseph during an early stage of his development. Chama’s psychological trauma is evidenced by her refusal to breastfeed and hold Joseph while she was in the hospital.

Chama’s death precipitates Joseph’s return to Kennedy, who has since cultivated a new familial arrangement. Within this narrative framework, an incident arises regarding the purported disappearance of Jennifer’s money, for which Joseph is unjustly scapegoated. This incident culminates in physical abuse from Kennedy. The film depicts Kennedy assaulting Joseph with a belt (Kennedy [Netflix 2022:00:23:15]), despite Joseph’s protestations of innocence, wherein he asserts, ‘I am not the one who stole the money’ (Joseph 23:23). Joseph’s innocence is later corroborated by the maid’s confession, who admits, ‘I am sorry, Joseph. It was your stepmother who made me do that. Otherwise, I didn’t want to do it’ (Maid [Netflix 2022:00:2:32]), revealing a conspiracy orchestrated by Jennifer.

Such incidents of both physical and psychological abuse illuminate the inhospitable environment to which children with albinism are subjected, particularly from their fathers. Research done in Nigeria bolsters these findings, indicating that fathers frequently emerge as perpetrators of violence against their children with albinism (Olagunju et al. 2024:8).

Moreover, Joseph grapples with the insidious effects of name-calling, which affect his psychological equilibrium. The term *Mwabi*, a derogatory Zambian noun connoting individuals with albinism, epitomises this harsh reality. Joseph was first called *Mwabi* when he sneaked out of the house to engage with his peers (A child from the playing ground [Netflix 2022:01:23:18]). He experiences ostracism, even from his half-siblings, who refuse to share a drink with him and ostracise him as a *Mwabi* (Mimi [Netflix 2022:00:26:34]). Additional names directed at Joseph include *Ghost* (Another child from the playing ground 1:23:19) and *Mwagoti* (The Man [Netflix 2022:01:05:33]). This ridicule is prevalent across African contexts, where children with albinism endure scorn while striving to integrate within their peer groups (Taylor & Lund 2008:1251). Research from Malawi found that people with albinism are called *pigs, white people*, and *makobiri* [literally, ‘money’] (Likumbo et al. 2021:5). In some countries, children with albinism are regarded as ghosts (Taylor et al. 2021:4) and *Shilumbu*, meaning ‘white person’ (Ngula 2023:434). *Mbunzu gozo* [black eater of manioc] is one of the names given to children with albinism in the Republic of Congo, while in Mali, they are called *gomble* [red man] (Harvagan & Chetty 2023:157; Reimer-Kirkham et al. 2022:722).

### Failing to live a normal life

Our study also found that children with albinism are systematically deprived of the opportunity to engage in a life analogous to that of their peers. A poignant moment transpired during the following dialogue between Joseph and his mother, Chama, and encapsulates this reality:

Joseph: ‘What time is Daddy coming?’

Chama: ‘Soon.’

Joseph: ‘So, I can go outside and play while I wait for him.’

Chama: ‘Come, sit here with me, I already told you, my boy, you are not well. But when you get better, you will go outside and play with your friends.’

Joseph: ‘But I don’t feel sick.’ (Netflix 2022:01:30:00–01:30:45)

There is also a broader limitation confronted by caregivers of children with albinism, who frequently navigate a life laden with constraints, sacrificing myriad opportunities (Likumbo et al. 2021:5). Chama’s life is eclipsed by her protective instincts towards Joseph, compelling her to eschew personal aspirations, including her partner Martin’s desire to expand their family. A telling exchange unfolded when Martin articulated his wish for more children:

Charma: ‘I am scared, Martin.’

Martin: ‘Scared of what?’

Charma: ‘I am scared that if we try for another, he will turn out to be just like Joseph. What if history repeats itself?’ (Netflix 2022: 00:49:05–00:48:42)

The condition of albinism, being intrinsically biological, remains bereft of curative options (Diallo et al. 2024:752). The fear surrounding the potential for another child with albinism can be real because there is evidence that some mothers would have two children with albinism (Reimer-Kirkham et al. 2022:727). In certain instances, this anxiety has culminated in the decision to terminate further pregnancies (Taylor et al. 2021:3).

### Bullying at school

On his inaugural day at school, Joseph encountered animosity from his classmates. Observations unveiled a girl spitting saliva into her armpit, an African cultural gesture emblematic of a wish to repel the prospect of birthing a child with albinism (Amato 2019). Likumbo et al. (2021:5) noted that some expectant mothers actively dissociate from children with albinism, fearing that proximity may transfer albinism to their unborn children. Furthermore, during a classroom seating allocation, Joseph was met with subtle rejection. The following is an excerpt from the interaction between the teacher (Madam), orchestrating a seat for Joseph, and Bernard, who was supposed to open the seat for him:

Bernard: ‘Sorry, Ma’am, this seat is taken.’

Madam: ‘By who?’

Bernard: ‘My bag.’

Madam: ‘Can your bag make room for Joseph here?’

Bernard: ‘I am sorry, it’s too heavy.’ [*We observed that Bernard was sarcastically pretending to lift his bag and eventually did not lift it from the chair*] (Netflix 2022:01:08:16–01:08:08)

This interplay serves to diminish Joseph’s standing, perpetuating a narrative that signifies he does not merit equal consideration within the classroom. Further instances of bullying ensued, illustrated by a subsequent encounter:

One of the classmates tells Joseph: ‘You really think you’re going to get away with that easily?’

Another classmate: ‘Oh, you want to cry?’

Joseph: ‘Please, just let me go.’

Another classmate: ‘Cry.’

Another classmate: ‘He’s going to cry.’ (Netflix 2022:01:02:01–01:01:54)

During the above conversation, we observed a change in Joseph’s demeanour, illustrated by his anguished expression, ultimately culminating in tears, which underscores the emotional toll he was under. The insensitivity and mockery from his peers serve to alienate him further, exacerbating feelings of isolation that may affect his self-esteem and academic performance.

### Violence and victimisation

Our findings illuminate the profound tribulations faced by Chama and Joseph, arising from deeply entrenched African superstitions, myths, stereotypes and cultural paradigms surrounding albinism. Joseph’s presence was mistakenly perceived as an augury of prosperity for entrepreneurial ventures. During an observance at a local tavern, a conversation erupted among patrons upon sighting Joseph returning from school:

‘The Man:‘Boss.’

The Boss:‘What?’

The Man: ‘Diamond outside.’

The Boss:‘What Diamond?’

The Man: ‘The Maggot, the white person born from a Black, can’t you see him?’

The Boss: ‘That’s a lot of money, my guy.’

The Man: ‘That’s a lot of money, especially for people like you.’ (Netflix 2022:01:05:42–01:05:27)

This pernicious belief culminated in an assault on Joseph, intended to enhance the tavern’s commercial viability. We observed a grievous injury inflicted upon his right leg, just below the patella, with a formidable blade (Netflix 2022:00:53:45). Body parts of children with albinism are often viewed as potent artefacts in rituals purported to engender wealth accumulation (Punaks et al. 2024:13; Reimer-Kirkham et al. 2022:723). Research conducted by Likumbo et al. (2021:5) indicates incidences of attempted genital mutilations targeting children with albinism in Malawi, a grim reality mirrored in Mozambique (Punaks et al. 2024:38; Taylor et al. 2021:2).

A testimony from a Zambian national further corroborates the existential perils faced by individuals with albinism, who remain perpetually vulnerable to life-threatening assaults and abductions (Punaks et al. 2024:28). Furthermore, there are alarming accounts of human remains being trafficked in black markets, (Harvagan & Chetty 2023:157; Reimer-Kirkham et al. 2022:721) where the entirety of a person with albinism may command prices soaring to $75 000.00, while individual body parts can fetch as much as $2000.00 (Misic 2024).

### Differential treatment

In Kennedy’s perception, Chama’s tribulations differ markedly from Jennifer’s. Despite Jennifer’s successful cohabitation with Kennedy while nurturing two children, Chama’s singular child, born with albinism, precipitated her eviction from the shared domicile. Kennedy’s prejudicial treatment of Chama stemmed from her association with a child with albinism. Akin to Joseph, who found himself denied the right to reside with his father, a right afforded to his siblings.

## Discussion

This section employs a human rights-based approach to the plethora of challenges besetting children with albinism and their mothers within African contexts, framing these challenges as a manifestation of human rights violations. According to Punaks et al. (2024:13), numerous fundamental rights are systematically infringed upon, children with albinism and their mothers. These violations permeate various societal strata, perpetrated by a diverse array of entities, including private organisations, institutional bodies, communal affiliations, familial units and individual actors (Reimer-Kirkham et al. 2022:721).

### Right to housing

A pivotal finding derived from the film underscores the disconcerting plight of a child with albinism and his mother, who were ousted from their home by the child’s father, resulting in a state of homelessness. Numerous accounts are documented in which fathers of children with albinism extricate themselves from their familial roles, as articulated by Taylor et al. (2021:5). Such circumstances undermine the right to housing conferred upon both children with albinism and their mothers, especially in instances where the father is the breadwinner.

Furthermore, the capitulation of the state to actualise the right to housing for children with albinism and their mothers constitutes a manifest infringement. Notably, Tanzania has instituted temporary shelters for persons with albinism (Punaks et al. 2024:40). Also, the government of Malawi epitomises a paradigm for other African nations through its state-sponsored housing initiatives designed for individuals with albinism. To date, Malawi has successfully erected 49 of the anticipated 67 residences intended for individuals with albinism throughout its jurisdiction (Banda 2024).

Loss of homes by children with albinism and their children contravene right to property enshrined in Article 14 of the African Charter on Human and People’s Rights (African Union 1981:5). Furthermore, Article 20 of the Protocol to the African Charter on the Rights of Persons with Disabilities (African Union 2018:18) affirms the right to an adequate standard of living, encompassing housing and social protection for children with disabilities, while Article 16 of the Protocol to the African Charter on the Rights of Women (African Union 2003:16) stipulates the right to housing for mothers of children with albinism.

The violation of housing rights for children with albinism manifests distinctly from that of their mothers. The paternal duty to provide shelter for a child with albinism is anchored in the overarching responsibilities inherent to parenthood, which endure beyond the dissolution of marriage, as articulated by Article 19 of the African Charter on the Rights and Welfare of the Child (African Union 1999:17). In contrast, mothers of children with albinism possess an innate right to reside in their matrimonial home throughout the duration of their unions, as well as to claim an equitable portion of the matrimonial home upon divorce or separation, as delineated in Article 7 of the Maputo Protocol to the African Charter on the Rights of Women (African Union 2003:9). Notably, Article 7(a) safeguards these mothers against arbitrary or discriminatory displacement by mandating adherence to formal legal procedures. This provision empowers mothers to assert their claims to property, sustenance or custody, thereby counteracting customs that unjustly impute misfortune upon women, particularly those burdened with children with albinism, thus compelling their departure from unions with scant recourse. Additionally, Article 7(e) fortifies the right to persist in the marital residence and extends legal protection against capricious marital dissolution. If a wife possesses a legal or customary entitlement to the familial dwelling, her expulsion without proper judicial process could precipitate a violation of her property rights.

### The right to dignity

It was one of the findings from the film that children with albinism are subjected to a duality of physical and psychological maltreatment. The case of Joseph epitomises the abuse manifesting in violence enacted by his father and peers within educational and communal spheres. This scenario serves as a microcosm of a broader, entrenched transgression against the rights and dignity of children with albinism. A particularly heinous incident transpired in Tanzania in May 2024, when a two-and-a-half-year-old girl was abducted in the Kagera region. This nefarious act involved an assault on the mother (Africa Albinism Network & Tanzania Albinism Society 2025:11). Such an incident encroaches upon the dignity of children with albinism, burdening them and their mothers with profound trauma. Around the same period in Geita, another region in Tanzania, a 10-year-old boy sustained injuries during an attack while engaged in the mundane task of fetching water (Africa Albinism Network & Albinism Multipurpose Organisation n.d.).

The jurisprudence surrounding the right to dignity for individuals with albinism was notably elucidated in the case of *Centre for Human Rights and others v United Republic of Tanzania* (African Court on Human and Peoples’ Rights 2025). Between 2000 and 2013, a tally of over 30 individuals with albinism in Tanzania were victims of violent transgressions (African Union: African Committee of Experts on the Rights and Welfare of the Child 2016:8). The Court adjudicated that the government’s dereliction in safeguarding individuals with albinism from predatory practices constituted inhuman and degrading treatment, thereby violating their dignity and freedom from torture. This judgement accentuates the obligations that African nations bear under the African Union’s human rights instruments to protect the dignity and other entitlements of individuals with albinism (Ayuk 2025). Misic (2024) posits that the investigation and prosecution of those culpable for assaults against individuals with albinism reinforces a commitment to justice and dignity for this vulnerable demographic.

Article 5 of the African Charter on Human and Peoples’ Rights categorically prohibits any manifestation of degrading treatment (African Union 1981:3). The African Commission on Human and Peoples’ Rights (2017:2) articulates that dignity is an inalienable attribute of every human being, with the nexus between the right to dignity and the unequivocal rejection of torture and ill-treatment being emphatically upheld. Furthermore, Article 8 of the Protocol to the African Charter on the Rights of People with Disabilities (Disability Protocol) distinctly underscores the necessity of safeguarding the dignity of children with albinism (African Union 2018:8), while Article 3 of the Maputo Protocol extends analogous protections to their mothers (African Union 2003:5). Article 16 of the African Charter on the Rights of the Child (African Union 1999:15) reinforces the imperative of shielding children from inhuman and degrading punishments, thereby preserving their inherent dignity.

The dignity of children with albinism is undermined through verbal abuse and name-calling. Such derogatory nomenclature contravenes a human rights-based paradigm and transgresses Article 28(2) of the Disability Protocol (African Union 2018:19), which mandates member states to ensure the preservation of identities, facilitating a full and dignified existence that promotes self-reliance and active societal participation. This verbal abuse not only erodes the authentic identities of individuals with albinism but also obstructs their right to exist devoid of stigmatising labels, adversely impacting their self-esteem.

In the African context, the notion of dignity is intricately intertwined with the concept of Ubuntu (humanism), which posits that an individual’s humanity is intrinsically linked to that of others (Gelaye 2021:123). Consequently, the perceptions and roles of community and familial structures in upholding dignity remain paramount (Gelaye 2021:123). The deleterious interactions encountered by children with albinism and their mothers within their communities can severely erode their dignity. The essence of human dignity, within a human rights-based framework, serves as a pivotal connective tissue binding all other rights to the individual.

According to Gelaye (2021:123):

In other words, when the individual loses his dignity, it is his human nature itself which is called into question, to the extent that it is likely to interrogate the validity of continuing to belong to human society … when dignity is lost, everything is lost. In short, when dignity is violated, it is not worth the while to guarantee most of the other rights. (p. 131)

Therefore, safeguarding the dignity of children with albinism and their mothers is indispensable within a human rights-based approach, as it is fundamentally linked to the intrinsic value of humanity, the foundational rationale for the assignment of human rights.

### The right to self-determination

The concept of self-determination is undermined for children with albinism and their maternal caregivers, whose lived experiences are permeated by an oppressive confinement rooted in pervasive anxieties. In the cinematic portrayal of Joseph, this confinement is vividly illustrated as he is confined to his mother’s domestic sphere. However, a more pervasive trend manifests across myriad African contexts, where children with albinism are relegated to state-sanctioned boarding schools or shelters. This institutionalised response effectively strips them of critical support from their local communities, thereby stifling their educational and social development (Africa Albinism Network & Albinism Multipurpose Organisation n.d.). In various African nations, temporally designated shelters have been instituted to ostensibly protect individuals with albinism from existential threats; for example, reports from 2016 reveal that Tanzania had 32 such facilities specifically for children with albinism (African Union: African Committee of Experts on the Rights and Welfare of the Child 2016:9). Among the 301 children residing in these centres, a demographic breakdown reveals 156 boys and 145 girls, with the youngest being merely 2 years old (African Union: African Committee of Experts on the Rights and Welfare of the Child 2016:9).

A seminal study by Franklin et al. (2018:4) involving interviews with 15 adult South Africans living with albinism elucidates that some of the detrimental outcomes of societal isolation are imposed by external environmental factors. In the same study, respondents reported challenges with self-perception and their intrinsic sense of belonging, both within their familial spheres and in the broader societal context. This marginalisation engenders a profound impediment to the cultivation of self-esteem among children with albinism, who are frequently rendered invisible within their communities (Franklin et al. 2018:4).

The onerous nature of this confinement constitutes a transgression of the inalienable right to self-determination, which empowers children with albinism and their mothers to navigate their existence with agency and autonomy. Article 20(1) of the African Charter on Human and Peoples’ Rights (African Union 1981) enunciates that:

All peoples shall have right to existence. They shall have the unquestionable and inalienable right to self-determination. They shall freely determine their political status and shall pursue their economic and social development according to the policy they have freely chosen. (p. 6)

Moreover, Article 14 of the Disability Protocol (African Union 2018:10) elucidates the right to inhabit a community of one’s preference on an equitable basis with others.

Self-determination encompasses the capacity for individuals to live autonomously, secure meaningful employment and cultivate relationships of their choosing (Franklin et al. 2018:5). However, this potential is significantly hampered by the isolation imposed on children with albinism, who are often compelled to languish within domestic confines (Franklin et al. 2018:5) or dwell in shelters (African Union: African Committee of Experts on the Rights and Welfare of the Child 2016:9). The bifurcation of mothers from the fathers of children with albinism exacerbates their predicament, compelling them to negotiate the delicate equilibrium between familial obligations and professional pursuits (Tokić, Ana Slišković & Ivanišević 2023:2). This struggle often precipitates an exodus from the workforce, as employment can be perceived as a distraction from maternal responsibilities towards their children with albinism (Tokić et al. 2023:2). Consequently, this withdrawal exacerbates their non-participation in consequential societal activities, encompassing work, religious observances and political engagement (Home 2002).

The pantheon of myths associated with albinism is both diverse and insidious, with each narrative contributing to the alienation of those affected. In the Democratic Republic of Congo, albinism is deemed an omen of misfortune, while in Kenya, it is inextricably linked to witchcraft (Punaks et al. 2024:35). These fallacies compel children with albinism to remain sequestered at home, with their mothers often sacrificing their own opportunities to ensure the safety of their children. Such confinement contravenes the right to self-determination, a fundamental prerogative that is indispensable for the holistic development of children with disabilities.

### Right to education

The fundamental right to education for children with albinism is systematically subverted across myriad dimensions. It was one of the findings in this study that the mother was harbouring her son from attending a formal school, predominantly because of concerns regarding potential victimisation. This sentiment reflects the pervasive societal attitudes in numerous African nations. In various instances, parents perpetuate violations of their children’s educational rights for disparate reasons; some may regard the financing of their education as an unwarranted expense (Adelakun & Ajayi 2020:245; Olagunju et al. 2024:8).

Joseph’s plight within the educational system unveils that he was subjected to egregious bullying. The integration of children with albinism into mainstream educational contexts is frequently obstructed by harassment, ubiquitous negative perceptions and a dearth of adequate health and educational provisions – phenomena tragically prevalent across various African countries, notably Zimbabwe (Punaks et al. 2024:12; Taylor & Lund 2008:1250). The absence of educational accommodations, including large-print textbooks, further exacerbates their educational predicament (Harvagan & Chetty 2023:161).

Educators, often entrenched in communities replete with myths and misconceptions about albinism, may possess apprehensions regarding their capability to proficiently instruct these learners (Franklin et al. 2018:6). Although minor adaptations within the classroom set-up are sometimes warranted, initiatives that educators could champion (Franklin et al. 2018:6), such measures are infrequent. Simple modifications, such as inscribing text in enlarged font on the chalkboard or seating those with albinism in a proximate vicinity to it, could significantly ameliorate their learning conditions (Likumbo et al. 2021:5).

The absence of educational accommodations, including large-print textbooks, further exacerbates their educational predicament (Harvagan & Chetty 2023:161).

Moreover, the scarcity of specialised institutions catering to the needs of children with albinism drastically constrains their educational accessibility. For example, Nigeria does not have albinism-friendly schools, a challenge that exists in most African nations (Olagunju et al. 2024:15). In instances where children with albinism are integrated into specialised learning establishments, the scope of their education often remains circumscribed, frequently eschewing critical subjects such as mathematics and the sciences (Punaks et al. 2024:28). Many of these specialised institutions may focus predominantly on visually impaired students, deploying braille, which further segregates children with albinism from their peers in mainstream educational settings (Punaks et al. 2024:28).

Challenges arising from the abysmal accommodations for individuals with albinism have culminated in approximately 10% of these children in Tanzania failing to transition to secondary education (Africa Albinism Network & Albinism Multipurpose Organisation n.d.). Legislative deficiencies regarding educational provisions for individuals with albinism are conspicuously manifest in various African jurisdictions. For instance, Tanzania’s *Education Act* does not stipulate the requisite provision of low-vision aids, magnifiers or large-print materials (Africa Albinism Network & Albinism Multipurpose Organisation n.d.). There exists a notable absence of regulatory mandates for educational institutions to supply essential materials pertinent to the needs of children with albinism, such as sunscreen, protective garments and prescription eyewear (Africa Albinism Network & Albinism Multipurpose Organisation n.d.).

Reports indicate that numerous children with albinism in Malawi are deprived of suitable educational institutions that offer special needs education. A child’s testimony, chronicled in a 2020 United Nations Africa Renewal study, underscores the ramifications of vision impairment, resulting in his slow academic progression (UN News: United Nations Africa Renewal 2020). While South Africa does proffer some specialised institutions for children with albinism (Franklin et al. 2018:6), a particular rural school narrowly constrained the utilisation of magnifiers and low-vision devices to select instructional segments, such as map reading (Franklin et al. 2018:6). In Zimbabwe and Zambia, children with albinism are frequently consigned to mainstream schools, where inclusion presents formidable challenges (Franklin et al. 2018:6).

The safety of children with albinism remains a concern, even amid their enrolment in specialised educational schools. In regions such as Malawi and Zambia, the concern for safety has compelled numerous guardians to eschew the attendance of their children at such institutions (Punaks et al. 2024:42). While certain boarding academies in diverse African contexts, like South Africa, may ostensibly proffer a bastion of refuge and educational access for children with albinism, these milieus frequently incite profound sentiments of isolation and loneliness, particularly during prolonged recesses when their peers retreat to families during holiday (Punaks et al. 2024:39). In Madagascar, despite the veneer of protective protocols purportedly extended by law enforcement, children with albinism often find themselves relegated to boarding facilities designed for the visually impaired, an occurrence decried by parents because of the consequential separation from their offspring (Punaks et al. 2024:36). Such disjunction from mainstream educational realms undermines the social assimilation of children with albinism within their communities (Adelakun & Ajayi 2020:245).

These diverse circumstances in violation of the right to education for children with albinism stand in glaring contrast to Article 17 of the African Charter on Human and Peoples’ Rights (African Union 1981:6), which unequivocally affirms the right to education as a human right. This principle is further bolstered by Article 16 of the Disability Protocol (African Union 2018:11), which mandates the provision of reasonable accommodations to ensure that children with albinism can fully actualise their educational rights. It is important to underscore that the right to education constitutes a fundamental tenet for every child’s development, irrespective of infirmity, as delineated in Article 11 of the African Charter on the Rights and Welfare of Children (African Union 1999:11).

The expansive meaning of educational rights encompasses opportunities for continuous learning throughout to adulthood. Mothers of children with albinism make remarkable sacrifices to support and care for their children, oftentimes at the detriment of their own educational pursuits. This phenomenon constitutes a transgression of a fundamental right, explicitly enshrined for women in Article 12(2) of the Maputo Protocol (African Union 2003:13). The systemic impediments confronted by these challenges accentuate the urgent necessity for comprehensive initiatives to ensure the safeguarding of educational rights and the enhancement of social inclusion for children with albinism and their mothers.

### The right to life

Our investigation reveals that the perilous existence of individuals with albinism is perpetuated by an array of myths and superstitious beliefs endemic to certain African cultures. These archaic notions have precipitated life-threatening circumstances for children with albinism, manifesting in heinous acts such as abduction and the harvesting of body parts (Punaks et al. 2024:13). The prevalence of ritualistic homicides targeting individuals with albinism is alarmingly high across numerous African nations. Tanzania documented 75 murders between 2000 and 2017, while Malawi recorded the tragic deaths of 20 individuals with albinism from 2014 to 2017 (Dixon 2017). Disturbingly, familial complicity has been observed, as exemplified in Malawi, where a biological sibling colluded with contract killers to assassinate his brother (BBC Media Action 2022). A particularly egregious case unfolded in Kagara, Tanzania, where a three-and-a-half-year-old girl was brutally murdered and mutilated, with her biological father among nine suspects implicated (Africa Albinism Network & Albinism Multipurpose Organisation n.d.). In other instances, traditional healers and close associates of those with albinism have orchestrated these atrocities (Dixon 2017). The assault on Joseph serves as a harrowing illustration of the tangible violence inflicted upon children with albinism, as they are often pursued or gruesomely disfigured for ritualistic purposes. This sequence of events reveals the profound threat to their fundamental right to live with safety.

While mothers may not be the primary targets of such heinous acts, their safety remains precarious; in their instinctual desire to safeguard their children, they risk becoming collateral casualties. Thus, the right to life for both children with albinism and their mothers is egregiously compromised. This systemic violation contravenes Article 4 of the African Charter on Human and Peoples’ Rights (African Union 1981:3), which enshrines the right to life for all Africans and prohibits the arbitrary deprivation of this right. The sanctity of life for children with albinism is further safeguarded by Article 5 of the African Charter on the Rights and Welfare of the Child (African Union 1999:9), and reinforced by Article 8 of the Disability Protocol (African Union 2018:8), which asserts that ‘every person with a disability has the inherent right to life and integrity’. Moreover, the lives of mothers of children with albinism are unequivocally protected by Article 4 of the Maputo Protocol (African Union 2003:6), which endows their lives with inherent value that must be respected and honoured. These provisions for the right to life of both children with albinism and their mothers are pervasive violations across the African continent, standing in stark contradiction to a human rights-based ethos that aspires to uphold the sanctity of life for all. The right to life constitutes a foundational tenet of the human rights paradigm, as the death of an individual obviates the possibility of exercising any further rights. An unyielding application of this human rights-based approach necessitates treating the right to life as sacrosanct and non-negotiable.

### Right to equality

The imperatives of equality and non-discrimination are axiomatic to the human rights-based approach. The pervasive inequities, stigmatisation and discrimination that beset children with albinism and their mothers warrant rigorous scrutiny. These individuals grapple with systemic injustices that are intricately intertwined with their visual impairments and dermal pigmentation, constituting egregious violations of their human rights (Punaks et al. 2024:12). The discriminatory practices they encounter are often predicated upon their skin colour, which is insidiously construed as a disability (Ibhawoh et al. 2022:2; Mswela 2018:26). Cultural superstitions and entrenched societal dogmas exacerbate the adversities faced by individuals with albinism, further entrenching their marginalisation (Reimer-Kirkham et al. 2022:721). The plight of these children is compounded by the pernicious loss of familial safeguarding, relegating them to a heightened state of vulnerability (Olagunju et al. 2024:9). Furthermore, their mothers disproportionately experience gender-based discrimination, which is exacerbated by their caregiving responsibilities, thereby circumscribing their access to socio-economic opportunities (Reimer-Kirkham et al. 2022:731).

These systemic inequities are unequivocally proscribed by Article 3 of the African Charter on Human and Peoples’ Rights (African Union 1981:3), which enshrines the right to legal protection sans discrimination. Concurrently, Article 3 of the African Charter on the Rights and Welfare of the Child (African Union 1999:9) delineates the imperative of equitable treatment for all children, irrespective of attributes such as disability, race or other distinguishing characteristics. Article 6 of the Disability Protocol (African Union 1999:9) underscores the need to implement proactive measures to promote equality for persons with disabilities. In light of this, it is incumbent upon African nations to undertake targeted interventions aimed at obliterating discriminatory practices against children with albinism, thereby ensuring their unimpeded access to the privileges and protections afforded to their counterparts. This advocacy resonates profoundly with the core tenets of a human rights-based approach, which necessitate the equal dispensation of rights, thereby requiring the equal enjoyment of rights for all children. Furthermore, African states are obligated to implement measures that empower mothers of children with albinism, thereby facilitating their attainment of rights commensurate with those of their male counterparts, as articulated in Article 2 of the Maputo Protocol (African Union 2003:6).

### Recommendations

African nations are urged to adopt a more vigorous human rights-based paradigm to advocate for the welfare of children with albinism and their mothers, enabling the full realisation of their rights. The right to housing, the construction of enduring residencies is both feasible and essential, particularly as children with albinism and their mothers represent a marginalised constituency. By adequately prioritising this demographic in national fiscal allocations, African countries could effectively fulfil these critical needs. The strategies employed by Kenya and Uganda, which emphasise the prevention of familial disintegration and the promotion of familial care, serve as paradigmatic examples that many African states have yet to adopt (Punaks et al. 2024:35, 42). This illustrates a valuable paradigm for other countries to develop frameworks that sustain the family unit for children with albinism.

To enhance the safety and dignity of children with albinism, each birth involving a child with this condition must be officially registered with local law enforcement. Such a measure necessitates an increase in police presence in communities frequented by these children, particularly within educational and recreational environments.

The right to education for children with albinism can be addressed through minimal yet significant adaptations in the classroom environment, including the use of enlarged print on boards and enabling these children to occupy front-row seating to optimise visibility. Furthermore, fostering a transformative ethos among educators and peers is imperative, achievable through awareness workshops conducted in schools that enrol children with albinism.

The integration of social workers is equally vital, as they can assist parents and family members in grasping the nuances of albinism through comprehensive counselling initiatives. This support system will empower children with albinism to flourish within a nurturing familial environment, where care and protection are paramount for their self-determination. Parents should be equipped with pertinent information regarding the genetics of albinism, preparing them for the potential of additional children with the condition if they choose to expand their families. Additionally, counselling in maternal and child health care settings can significantly enhance medical literacy.

The violence and acts of aggression directed at children with albinism are perpetuated mainly by a pervasive ignorance surrounding the disability. A regional strategy dedicated to disseminating accurate information about albinism through a variety of channels, including African media outlets, advocacy initiatives and social media platforms, is imperative. Regional symposia should also be convened to raise awareness of albinism and promote the exchange of knowledge regarding this condition. These gatherings must prioritise the inclusion of individuals with albinism, allowing them to share their experiences and propose constructive interventions to mitigate human rights violations.

Ibhawoh et al. (2022:4) attribute the discrimination faced by children with albinism and their mothers to the inadequate enforcement of existing human rights instruments throughout Africa. Nevertheless, given the distinctive and intersectional nature of the human rights infringements encountered by these individuals, reliance on conventional human rights frameworks proves insufficient (Ibhawoh et al. 2022:7). Consequently, we advocate for the establishment of a comprehensive human rights framework tailored to the unique plight of children with albinism and their mothers, addressing these multifaceted human rights abuses holistically.

## Conclusion

The myriad challenges confronting children with albinism and their mothers, as depicted in the movie ‘Can You See Us’, resonate profoundly with those encountered in numerous African nations. These adversities starkly illustrate that children with albinism and their mothers are perilously distanced from enjoying their rights comparably to their counterparts. These entitlements are explicitly enshrined within various African legal frameworks. The rights currently being transgressed encompass housing, dignity, self-determination, education, life and equality. The extant regional action plan is demonstrably inadequate in offering or integrating a thorough human rights-based approach, thereby failing to ensure that all individuals, especially vulnerable groups, are accorded fundamental rights. To achieve the aspirational goals inherent within a human rights framework, there is an imperative to ameliorate housing conditions, enhance the visibility of law enforcement in locales housing children with albinism, adapt educational environments to their needs, implement awareness campaigns, facilitate counselling services by adept social workers for families and devise a specialised regional framework to safeguard the rights of children with albinism and their mothers.

## References

[CIT0001] Adelakun, O.S. & Ajayi, M.A.O., 2020, ‘Eliminating discrimination and enhancing equality: A case for inclusive basic education rights of children with albinism in Africa’, *Nigerian Journal of Medicine* 29(2), 244–251. 10.4103/NJM.NJM_50_20

[CIT0002] Africa Albinism Network & Albinism Multipurpose Organisation, n.d., *Shadow Report to the Committee on the Rights of Persons with Disabilities for the Review of Zambia: Report on the situation of persons with albinism in Zambia*, viewed 14 October 2025, from https://africaalbinismnetwork.org/wp-content/uploads/2024/02/Report-on-the-situation-of-persons-with-albinism-in-Zambia-to-the-CRPD-12.02.2024.pdf.

[CIT0003] Africa Albinism Network & Tanzania Albinism Society, 2025, *Joint alternative report on the human rights situation of children with albinism in Tanzania submitted to the committee on the rights of the child*, viewed 13 October 2025, from https://africaalbinismnetwork.org/wp-content/uploads/2025/03/Joint-Alternative-Report-on-the-Human-Rights-Situation-of-Children-with-Albinism-in-Tanzania.pdf.

[CIT0004] African Commission on Human and Peoples’ Rights, 2017, *General comment no. 4 on the African charter on human and peoples’ rights*, viewed 31 March 2025, from https://policehumanrightsresources.org/content/uploads/2021/07/achpr_general_comment_no._4_english.pdf?x49094.

[CIT0005] African Court on Human and Peoples’ Rights, 2025, *Centre for human rights and others v united republic of Tanzania, Application 019/2018*, viewed 14 October 2025, from https://www.african-court.org/cpmt/storage/app/uploads/public/67a/352/f2e/67a352f2e80cc613846453.pdf.

[CIT0006] African Union, 1981, *African charter on human and peoples’ rights*, viewed 31 March 2025, from https://au.int/sites/default/files/treaties/36390-treaty-0011_-_african_charter_on_human_and_peoples_rights_e.pdf.

[CIT0007] African Union, 1999, *African charter on the rights and welfare of the child*, viewed 31 March 2025, from https://au.int/sites/default/files/treaties/36804-treaty-african_charter_on_rights_welfare_of_the_child.pdf.

[CIT0008] African Union, 2003, *Protocol to the African charter on human and peoples’ rights on the rights of women in Africa*, viewed 31 March 2025, from https://www.au.int/en/treaties/protocol-african-charter-human-and-peoples-rights-rights-women-africa.

[CIT0009] African Union, 2018, *Protocol to the African charter on human and peoples’ rights on the rights of persons with disabilities in Africa*, viewed 31 March 2025, from https://au.int/sites/default/files/treaties/36440-treaty-protocol_to_the_achpr_on_the_rights_of_persons_with_disabilities_in_africa_e.pdf

[CIT0010] African Union: African Committee of Experts on the Rights and Welfare of the Child, 2016, *Report on investigative mission on the situation of children with albinism in temporary holding shelters – Tanzania*, viewed 15 October 2025, from https://www.acerwc.africa/sites/default/files/2023-02/Investigative_Mission_on_the_Situation_of_Children_with_Albinism_pages.pdf.

[CIT0011] Amato, B., 2019, *Albinism inside out*, viewed 26 June 2025, from https://www.wits.ac.za/news/latest-news/research-news/2019/2019-11/albinism-inside-out.html.

[CIT0012] Ayuk, B., 2025, ‘African Court rules in favour of Persons with Albinism, orders Tanzania to take concrete action’, viewed 10 October 2025, from https://www.ihrda.org/2025/02/african-court-rules-in-favour-of-persons-with-albinism-orders-tanzania-to-take-concrete-action/.

[CIT0013] Banda, E.M., 2024, ‘Construction of houses for people with albinism on track – government’, *Malawi24*, viewed 14 April 2026, from https://malawi24.com/2024/07/12/construction-of-houses-for-people-with-albinism-on-track-government/.

[CIT0014] Benyah, F., 2017, ‘Equally able, differently looking: Discrimination and physical violence against persons with albinism in Ghana’, *Journal for the Study of Religion* 30(1), 161–188. 10.1007/978-3-031-40754-3_33

[CIT0015] Broberg, M. & Sano, H.-O., 2017, ‘Strengths and weaknesses in a human rights-based approach to international development – An analysis of a rights-based approach to development assistance based on practical experiences’, *The International Journal of Human Rights* 22(5), 664–680. 10.1080/13642987.2017.1408591

[CIT0016] Buyco, M., Reimer-Kirkham, S., Astle, B., De Waal, M., Kromberg, J., Mazibuko, N. et al., 2025, ‘Access to healthcare by mothers impacted by albinism in South Africa’, *Southern Africa Journal on Albinism Economic and Social Rights Southern Africa* 2025, 12–15.

[CIT0017] BBC Media Action, 2022, *Malawi men jailed over murder of man with albinism*, viewed 14 April 2026, from https://www.bbc.com/news/world-africa-61959875.

[CIT0018] Chacon-Hurtado, D., Kazerounian, K., Hertel, S., Mellor, J., Barry, J.J. & Ravindran, T., 2024, ‘Engineering for human rights: The theory and practice of a human rights–based approach to engineering’, *Science, Technology, & Human Values* 19(4), 898–934. 10.1177/01622439231211112

[CIT0019] Chagutah, T., 2025, ‘Southern Africa Journal on albinism economic and social rights southern Africa’, *Journal on Albinism Economic and Social Rights* 6–7, viewed 14 April 2026, from https://www.amnesty.org/en/wp-content/uploads/2025/06/AFR0194872025ENGLISH.pdf.

[CIT0020] Chirwa, M. & Kalaluka, L., 2025, *Albinism and rights in Zambia: An exploration of experiences, sociocultural conditions and access to justice’ study report*, viewed 14 April 2026, from https://www.unicef.org/zambia/media/7411/file/Albinism-and-Rights-Final-September.pdf.pdf#:~:text=The%20Act%20prohibits%20discrimination%20against,on%20an%20equal%20basis%20with.

[CIT0021] Diallo, M., Sylla, O., Sidibé, M.K., Plaisant, C., Mercier, E., Sequeira, A. et al., 2024, ‘Genotypic spectrum of albinism in Mali’, *Pigment Cell & Melanoma Research* 37(6), 752–761. 10.1111/pcmr.13175 38720644

[CIT0022] Dixon, R., 2017, ‘In parts of Africa, people with albinism are hunted for their body parts. The latest victim: A 9-year-old boy’, *Los Angeles Times*, 15 June, viewed 10 May 2025, from https://www.latimes.com/world/africa/la-fg-malawi-albinos-hunted-2017-story.html.

[CIT0023] Ero, I., Muscati, S., Boulanger, A.-R. & Annamanthadoo, I., 2021, ‘People with albinism worldwide: A human rights perspective’, viewed 12 December 2025, from https://www.ohchr.org/sites/default/files/Documents/Issues/Albinism/Albinism_Worldwide_Report2021_EN.pdf.

[CIT0024] Franklin, A., Lund, P., Bradbury-Jones, C. & Taylor, J., 2018, ‘Children with albinism in African regions: Their rights to “being” and “doing”’, *BMC International Health and Human Rights* 18, 2. 10.1186/s12914-018-0144-8 29329540 PMC5767025

[CIT0025] Gelaye, T.A., 2021, ‘The role of human dignity in the “human rights” jurisprudence of the African commission on human and peoples’ rights’, *African Human Rights Yearbook* 5(1), 116–134. 10.29053/2523-1367/2021/v5a6

[CIT0026] Hargovan, H. & Chetty, R., 2023, ‘The lived experiences of persons with albinism in the Northern Cape, South Africa’, *African Journal of Social Work* 13(3), 155–166. 10.4314/ajsw.v13i3.4

[CIT0027] Home, A., 2002, ‘Challenging hidden oppression: Mothers caring for children with disabilities’, viewed 31 March 2025, from https://ojs.uwindsor.ca/index.php/csw/article/view/5652/4620.

[CIT0028] Ibhawoh, B., Reimer-Kirkham, S., Ero, I., Mgijima-Konopi, I., Beaman, L., Senkoro, P. et al., 2022, ‘Shifting wrongs to rights: Lessons in human rights from the situation of mothers impacted by albinism in Africa’, *Journal of Human Rights Practice* 14(3), 838–858. 10.1093/jhuman/huac038

[CIT0029] Johnson, S., 2023, ‘“A fire inside me”: The Zambian singer who overcame prejudice to change attitudes to albinism’, *The Guardian*, 13 September, viewed 08 May 2025, from https://www.theguardian.com/global-development/2023/sep/13/a-fire-inside-me-the-zambian-singer-who-overcame-prejudice-to-change-attitudes-to-albinism#:~:text=Chiti’s%20father%20divorced%20his%20mother,it%20difficult%20to%20fit%20in.

[CIT0030] Likumbo, N., De Villiers, T. & Kyriacos, U., 2021, ‘Malawian mothers’ experiences of raising children living with albinism: A qualitative descriptive study’, *African Journal of Disability* 10, a693. 10.4102/ajod.v10i0.693 PMC8063528 33937005

[CIT0031] Malawi News Agency, 2024, ‘People with albinism hail housing project’, viewed 08 May 2025, from https://lands.gov.mw/index.php/our-projects.

[CIT0032] Misic, L., 2024, ‘How the albino myth is endangering Tanzanian children’, viewed 13 October 2025, from https://www.humanium.org/en/how-the-albino-myth-is-endangering-tanzanian-children/.

[CIT0033] Mswela M.M., 2022, ‘Living with Albinism in South Africa: Uncovering the Health challenges from a legal perspective’, *Orbiter* 43(1), 25–48.

[CIT0034] Mswela, M., 2025, ‘Southern Africa Journal on albinism economic and social rights southern Africa’, *Journal on Albinism Economic and Social Rights* 24–30, viewed n.d., from https://www.amnesty.org/en/wp-content/uploads/2025/06/AFR0194872025ENGLISH.pdf.

[CIT0035] Mswela, M.M., 2018, ‘Does albinism fit within the legal definition of disability in the employment context? A comparative analysis of the judicial interpretation of disability under the SA and the US non-discrimination laws’, *Potchefstroom Electronic Law Journal* 21, 1–37. 10.17159/1727-3781/2018/v21i0a1684

[CIT0036] Mtonga T., Lungu F, Kalimaposo K. & Mandyata J., 2025, ‘Education now: The role of families in the education of learners with albinism in Zambia’, *Southern Africa Journal on albinism Economic and Social Rights* 67–75.

[CIT0037] Netflix, 2022, ‘Can you see us?’, viewed 31 March 2025, from https://www.netflix.com/watch/81682802?trackId=268410292&tctx=0%2C0%2C5572aa66-b56a-4570-9345-6531551f957d-14382030%2C5572aa66-b56a-4570-9345-6531551f957d-14382030%7C2%2Cunknown%2C%2C%2CtitlesResults%2C%2CVideo%3A81682802%2CdetailsPagePlayButton.

[CIT0038] Ngula, A., 2023, ‘The power of information and coping with albinism: An autoethnographic study’, *IFLA Journal* 49(2), 432–442. 10.1177/0340035222110389

[CIT0039] Olagunju, A., Mabhala, M., Buck, G. & Taylor, L., 2024, ‘Being different: What it means to be a person with albinism in Nigeria’, *Disability & Society* 40(7), 1872–1896. 10.1080/09687599.2024.2380502

[CIT0040] Pane, E. & Yanis, T.Z.A., 2023, ‘Affirmative policy a necessity for fulfilling the political rights of persons with disabilities’, *Constitutionale* 4(2), 147–158. 10.25041/constitutionale.v4i2.3164

[CIT0041] Punaks, M., Baker, C. & Miti-Drummond, M.A., 2024, ‘OHCHR 2023 children with albinism and care identifying family and community-based care solutions for children with albinism’, viewed 10 May 2025, from https://www.ohchr.org/sites/default/files/2025-02/a-79-175-annex.pdf.

[CIT0042] Rais, F.M, & Fadillah, D., 2025, ‘Semiotic Analysis of Roland Barthes on the Lyrics of “HOPE” by XXXTENTACION’, *Jurnal Audiens* 6(1), 1–10. 10.18196/jas.v6i1.459

[CIT0043] Reimer-Kirkham, S., Astle, B., Ero, I., Imafidon, E. & Strobell, E., 2022, ‘Mothering, albinism and human rights: The disproportionate impact of health-related stigma in Tanzania’, *Foundations of Science* 27, 719–740. 10.1007/s10699-020-09701-0

[CIT0044] Reimer-Kirkham, S., Astle, B., Kromberg, J., Mgijima-Konopi, I., Shirley Mooa, R., De Waal, M. et al., 2024, ‘Birth stories of South African mothers of children with albinism: A critical human rights analysis’, *International Journal of Africa Nursing Sciences* 20, 100650. 10.1016/j.ijans.2023.100650

[CIT0045] Reimer-Kirkham, S., Ero, I., Mgijima-Konopi I., Strobell, E. & Astle, B., 2021, ‘Mothering and albinism: Recommendations for disability rights in Africa’, *African Disability Rights Yearbook* 9, 283–292. 10.29053/2413-7138/2021/v9a14

[CIT0046] Strobell, E., 2020, ‘Exploring the experience of mothers who have children with albinism in Tanzania: A critical ethnography’, Masters thesis, Trinity Western University, pp. 1–289.

[CIT0047] Taylor, J., Bradbury-Jones, C., Ogik, P., Kawuma, F., Betts, J. & Lund, P., 2021, ‘Reactions to and explanations for the birth of a baby with albinism: A qualitative study in Busoga, Uganda’, *BMJ Open* 11, e040992. 10.1136/bmjopen-2020-040992 PMC7907870 33622943

[CIT0048] Taylor, J.S. & Lund, P., 2008, ‘Experiences of a feasibility study of children with albinism in Zimbabwe: A discussion paper’, *International Journal of Nursing Studies* 45, 1247–1256. 10.1016/j.ijnurstu.2007.05.009 17602690

[CIT0049] United Nations, 1948, *Universal declaration of human rights*, viewed 14 April 2026, from https://www.refworld.org/legal/resolution/unga/1948/11563.

[CIT0050] Tokić, A., Ana Slišković, A. & Ivanišević, M.N., 2023, ‘Well-being of parents of children with disabilities – Does employment status matter?’, *Social Sciences* 12(8), 463. 10.3390/socsci12080463

[CIT0051] UN News: United Nations Africa Renewal, 2020, *Making education safe for children with albinism in Malawi*, viewed 13 October 2025, from https://africarenewal.un.org/en/magazine/making-education-safe-children-albinism-malawi.

